# Association between vitamin D and development of otitis media: A PRISMA-compliant meta-analysis and systematic review

**DOI:** 10.1097/MD.0000000000004739

**Published:** 2016-10-07

**Authors:** Hong-Bo Li, Xu-Hui Tai, Yue-Hong Sang, Jian-Ping Jia, Zhen-Ming Xu, Xiao-Feng Cui, Song Dai

**Affiliations:** Department of Otolaryngology, Head & Neck Surgery, No. 463 Hospital of Chinese PLA, Shenyang, Liaoning, China.

**Keywords:** meta-analysis, otitis media, vitamin D

## Abstract

**Background:**

Nutrients related to serum vitamin D level were previously shown to be significantly associated with the risk of many chronic diseases. This study aimed to assess potential relationships between serum vitamin D level and otitis media (OM) risk.

**Methods:**

PubMed, EMBASE, and Cochrane Library databases were searched till Aug 18, 2015 for studies of quantitative OM risk estimates in relation to serum vitamin D level. The odds ratio and weighted mean difference, with 95% confidence intervals (CIs), were used to measure the relationship between serum vitamin D level and OM risk.

**Results:**

Of the 89 articles identified by database search, 5 studies reported data of 16,689 individuals were included in our meta-analysis. We noted participants with OM was associated with lower level of plasma vitamin D when compared with patients without OM (weighted mean difference −5.67; 95% CI −8.08 to −3.26, *P* < 0.001). Furthermore, as compared with control group, serum vitamin D level was not associated with the risk of OM (odds ratio 0.80, 95% CI 0.47–1.38, *P* = 0.425). Subgroup analyses suggested that participants with acute OM might associate with lower serum vitamin D level.

**Conclusions:**

Plasma vitamin D level might play an important role on the progression of acute OM, whereas no significant impact in patients with chronic OM.

## 1 Introduction

In terms of acute otitis media (AOM), chronic otitis media (COM), and otitis media with effusion (OME), otitis media (OM) refer to inflammation of the middle ear, which are most common pediatric diseases and the leading cause of visiting pediatricians.^[1,2]^ Both in developed and developing countries, the incidence of AOM in children is high.^[3]^ Usually, AOM caused by acute viral upper respiratory tract infection is a complication of Eustachian tube dysfunction.^[4]^ Once children suffer from recurrent episodes of AOM (rAOM) and long-term secretory otitis media (SOM), these diseases are more inclined to develop into COM.^[5]^

Currently, the treatment for AOM mainly depends on antibiotic therapy. In consideration of a major public health challenge for antibiotic-resistant bacteria, more attentions need to be paid to improving the management of AOM, which involves postponing antibiotic therapy.^[4]^ Although most studies showed a reduction in the risk of new AOM episodes in children with a history of rAOM, none of the treatment is completely effective.^[6]^

Recent studies related to serum 25-hydroxy vitamin D (25[OH] VD) showed that vitamin D might play a strong immunomodulatory role in improving the incidence and severity of bacterial and viral infections.^[6]^ In addition, several studies suggested that children with low serum 25(OH) VD levels suffer from respiratory infectious diseases at a high risk.^[7–12]^ Theoretically, the lack of vitamin D could cause an increased risk of rAOM, and vitamin D supplementation could be related to the limitation of the number of new episodes in OM-prone children. To evaluate the relationship between vitamin D and the risk of OM, we performed a meta-analysis and systemic review of current relevant studies and trials.

## 2 Materials and method

This review was conducted and reported according to the Preferred Reporting Items for Systematic Reviews and Meta-Analysis Statement issued in 2009 (Checklist S1).^[13]^ Ethics approval was not necessary for this study, as only de-identified pooled data from individual studies were analyzed.

### 2.1 Definitions

Otitis media, as a group of inflammatory diseases of the middle ear, includes 3 main types (AOM, OME, and chronic secretory otitis media [CSOM]). To differentiate between AOM, OME, and CSOM, it is important to confirm the diagnosis of OM, and bulging of the tympanic membrane is the best sign to differentiate AOM from OME.

25-hydroxyvitamin D, also known as calcifediol or calcidiol, is produced by hydroxylation of vitamin D_3_.^[14]^ The normal range of 25(OH) VD varies widely depending on several factors, such as age and geographic location.

### 2.2 Data sources and search strategy

A systemic review of the literature was performed in PubMed, EMBASE, and Cochrane library for all observational studies regarding the association between vitamin D and OM without any restrictions to languages and calendar date.

Furthermore, a total of 89 articles were identified using the following terms: (“otitis media” or “tympanitis” or “acute otitis media” or “AOM” or “adhesive otitis media” or “OME” or “catarrhal otitis media” or “catarrh tympanitis” or “middle ear inflammation”) AND (“vitamin D” or “ergocalciferol” or “calciferol” or “cholecalciferol”). To further search relevant studies, the reference lists from the retrieved studies were manually reviewed. All the included articles were reviewed by 2 authors independently for inclusion evaluation. Any inconsistencies between these 2 authors were settled down by the primary author until a consensus was reached.

### 2.3 Eligibility criteria

All studies with full texts to investigate the association between vitamin D and OM or the vitamin D level in patients with OM were included. Within the articles, although the age of participants was not limited, their disease status included AOM, rAOM, COM, or CSOM. The literature search and study selection was independently undertaken by 2 authors, and any inconsistency was settled down by group discussion until a consensus was reached. The study was eligible for inclusion if the study investigated the relationship between vitamin D and the progression of OM, and the study reported effect estimate or the levels of plasma vitamin D in case and control group.

### 2.4 Data extraction and statistical analysis

Data extraction was done by the first author and checked by the second. The extracted data included: study design, country and published year, age of participants, sample size, disease status, percentage of male, and blood vitamin D level. We examined the association between vitamin D levels and progression of OM on the basis of event/sample size in each group or mean, standard deviation, and sample size in case and control group. Weighted mean difference (WMD) or odds ratio (OR) and corresponding 95% confidence intervals (CIs) were evaluated by using the outcomes extracted from each study before data pooling. WMD with 95% CI was employed to calculate the level of vitamin D in case and control group, whereas OR and 95% CI were used to calculate the effect of vitamin D status on the risk of OM. Plasma vitamin D levels were considered sufficient if they were greater than 20 ng/mL. Heterogeneity between studies was examined using the Q statistic, and the *P* value less than 0.10 was regarded as significant heterogeneity.^[15,16]^ If *P* value was less than 0.10, the random-effect model was used, otherwise the fixed-effect model was employed.^[17,18]^ Sensitivity analyses were conducted by removing each individual study from the pooled analysis.^[19]^ Subgroup analyses were also conducted on the basis of disease status and mean age. In the planning stage, publication bias tests were conducted, whereas few studies reported the relationship between plasma vitamin D level and the progression of OM. The results were variable and unreliable (data not shown). All reported *P* values were 2-sided, and *P* value less than 0.05 was regarded as statistically significant for summary results. Statistical analyses were conducted by using STATA software (version 10.0; Stata Corporation, College Station, TX).

## 3 Results

**Figure 1:**
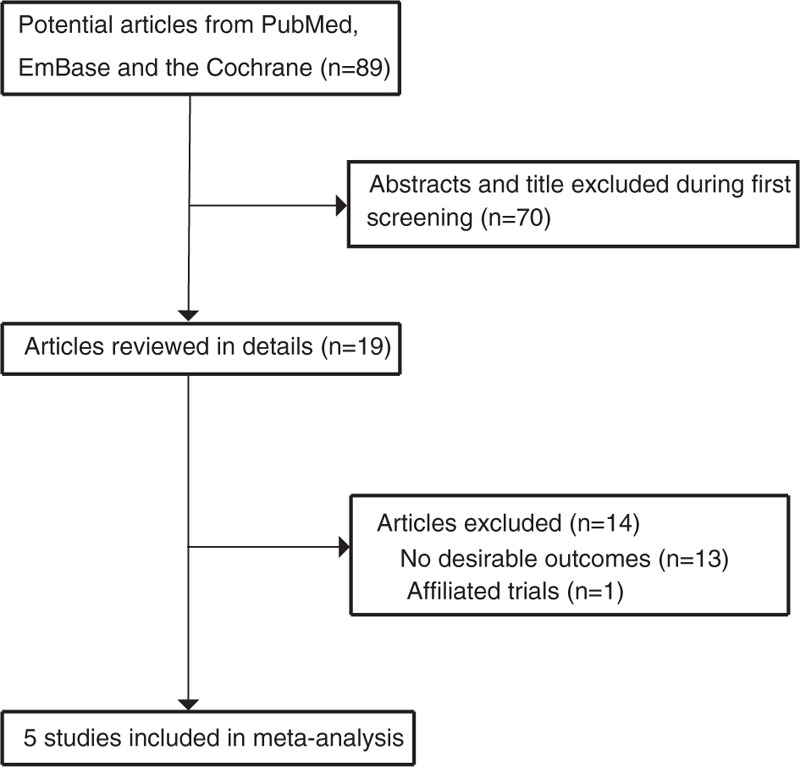
Flow diagram of the literature search and trials selection process.

The study selection process was shown in Fig. 1. We identified 89 articles in our initial search, of which 70 were excluded after duplicates, and irrelevant studies were excluded. A total of 19 potentially eligible studies were selected. After detailed evaluations, 5 studies were selected for the final meta-analysis.^[6,20–23]^ A manual search of the reference lists of these studies did not yield any new eligible studies. The general characteristics of the included studies are presented in Table 1.

**Table 1:**
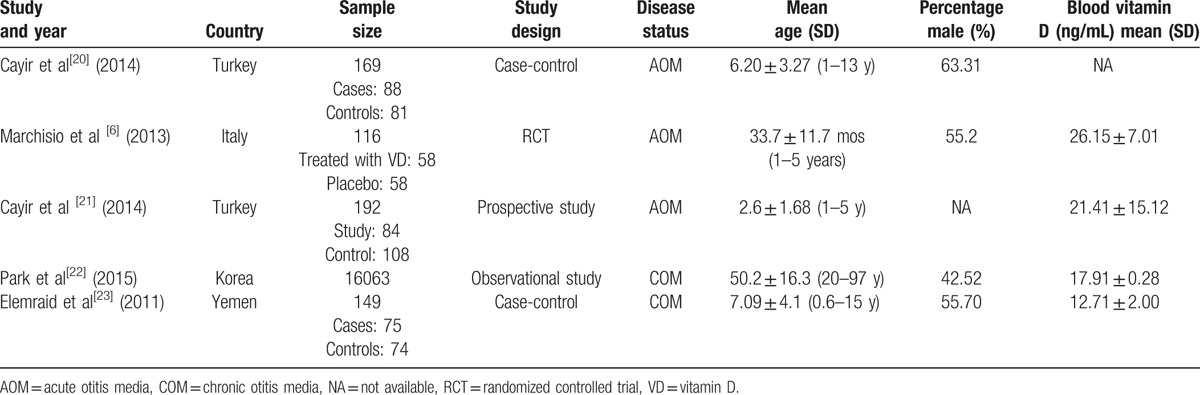
Baseline characteristic of studies included in the meta-analysis.

Of the 5 included studies (for a total of 16,689 individuals), 1 was a randomized controlled trial (RCT),^[6]^ 1 was a prospective cohort study,^[21]^ 2 were case-control studies,^[20,23]^ and the remaining 1 was a observational study.^[22]^ The sample size ranged from 116 to 16,063, and the age ranged from 2.6 to 50.2 years. Three studies were conducted in Europe,^[6,20,21]^ and the remaining 2 were conducted in Asia.^[22,23]^

**Figure 2:**
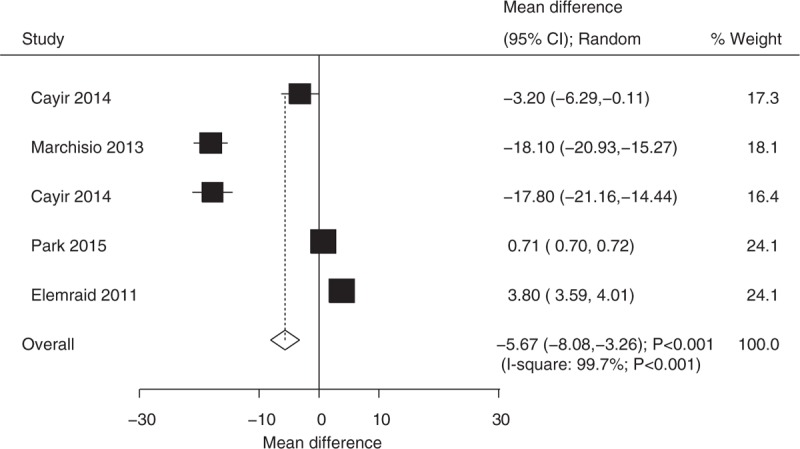
Relationship between plasma vitamin D level and the risk of OM. OM = otitis media.

A total of 5 studies reported mean and SD for plasma vitamin D levels in case and control group.^[6,20–23]^ Cayir et al and Marchisio et al found OM patients were associated with lower level of plasma vitamin D as compared with control group.^[6,20,21]^ However, Elemraid et al^[23]^ suggested participants with OM were associated with higher level of plasma vitamin D when compared with control group. Finally, Park et al^[22]^ suggested there was no significant difference between patients with OM and control for level of plasma vitamin D. In summary, the pooled WMD indicated that participants with OM was associated with lower level of plasma vitamin D level (WMD −5.67; 95% CI −8.08 to −3.26, *P* < 0.001; random-effect model; Fig. 2). Substantial heterogeneity was observed across included studies, sensitivity analysis was conducted, and after each study was sequentially excluded, the conclusion was not affected. Subgroup analyses suggested patients with AOM were associated with lower level of plasma vitamin D, whereas no significant impact was observed in other subsets (Table 2).

**Table 2:**
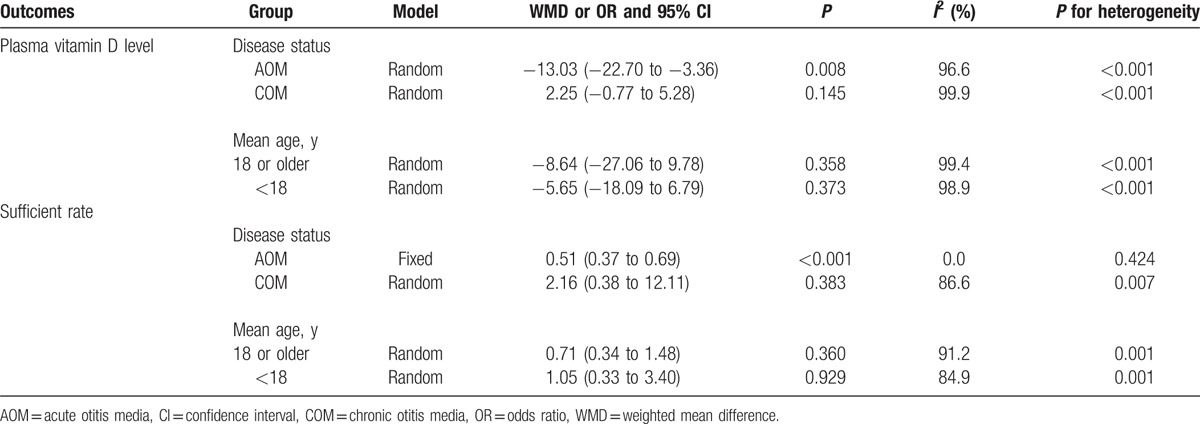
Subgroup analyses.

**Figure 3:**
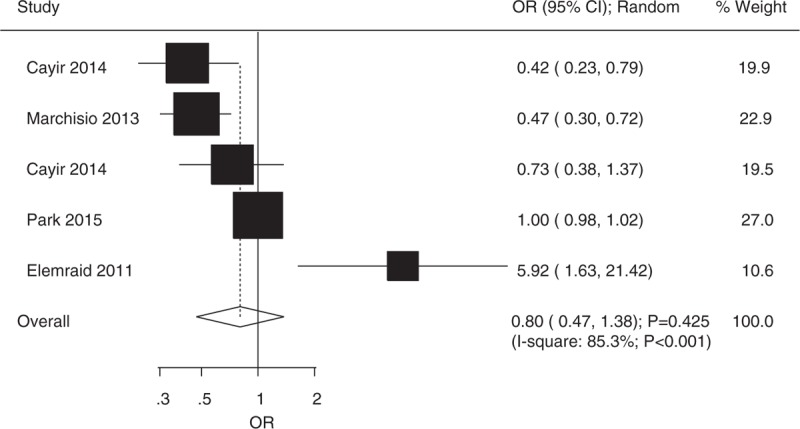
Relationship between number of participants with sufficient vitamin D level and the risk of OM. OM = otitis media.

A total of 5 studies reported number of participants with sufficient vitamin D levels in case and control group.^[6,20–23]^ Cayir et al^[20]^ and Marchisio et al^[6]^ suggested that patients with OM were associated with lower number of participants with sufficient vitamin D levels, whereas Elemraid et al^[23]^ indicated that participants in case group showed higher number of participants with sufficient vitamin D level when compared with those in control group. The summary OR revealed that plasma vitamin D status had no significant effect on OM (OR 0.80, 95% CI 0.47–1.38, *P* = 0.425; random-effect model; Fig. 3). Although substantial heterogeneity was detected, after each study was sequentially excluded from the pooled analysis, the conclusion was not affected by the exclusion of any specific study. Subgroup analyses indicated patients with AOM were associated with lower number of participants with sufficient vitamin D level (Table 2).

## 4 Discussion

This meta-analysis and systemic review was based on 2 case-control studies, 1 RCT, 1 observational study, and 1 prospective study, and provided up-to-date evidence for the risk of OM (AOM and COM) in association with vitamin D levels. A total 16,689 individuals in this quantitative study included a board range of populations. The main finding of our meta-analysis was that patients with OM was associated with lower level of plasma vitamin D. Cayir et al^[20]^ and Marchisio et al^[6]^ also found that a higher incidence of OM was frequently seen in patients with vitamin D deficiency, especially in winter. Additionally, vitamin D hypovitaminosis was common in children who suffered from rAOM and was associated with an increased number of AOM patients. Furthermore, it is efficacious for patients to use vitamin D as an adjuvant therapy in the treatment of upper and lower respiratory infections such as OM. Whereas Elemraid et al^[23]^ reported contradictory results.

There has been no meta-analysis study evaluating the relationship between vitamin D level and OM. Similarly, in a systematic review by Elemraid et al,^[24]^ there were no human study data reported on vitamin D and middle-ear diseases. Conversely, another previous review showed that a lower serum 25(OH)D level in children with rAOM was observed compared with that in healthy group, and argued that vitamin D supplementation could significantly reduce the incidence of new AOM episodes.^[2]^ However, the association between serum 25(OH)D levels and the risk of OM has not been fully explored. Furthermore, the optimal dosage of vitamin D for reducing the occurrence of AOM episodes still needs to be determined. Finally, this review only discussed the AOM in children (without adults) and other types of OM. As a result, we conducted a meta-analysis and systemic review to evaluate the relationship between vitamin D levels and of the risk of OM.

Among these included studies, 3 of them enumerated that active vitamin D was a major immunomodulator and could induce the antimicrobial peptides such as cathelicidin and defensin, and then increased natural defenses and reduced risk of infections.^[6,20,21]^ Similarly, in our current study, there was no significant increase in the incidence of new episodes of OM (AOM, rAOM, CSOM, and COM) in both children and adults with vitamin D hypovitaminosis or lower serum 25(OH)D levels. However, the standardization of a lower serum 25(OH)D level among different laboratories did not achieve an unified standard because of several factors, including age and geographic locations.^[25]^ In addition, another study presented a different point of view that vitamin D hypovitaminosis was not the main risk factor of CSOM, although vitamin D deficiency was correlated with CSOM morbidity and disease severity.^[23]^ In other words, it was unclear whether low levels of serum 25(OH)D was associated with the risk of OM infection or disease severity.^[26]^ The different results between Elemraid et al^[23]^ and us could be due to vitamin D deficiency caused by local dietary or therapeutic origin, or due to the fact that our study included all types of OM instead of only CSOM.

Our study demonstrated that vitamin D might be an effective adjuvant therapy in the treatment of OM. Meanwhile, the included trials showed significantly low serum 25(OH)D levels in both children and adults with recurrent OM. Previous studies suggested that it was efficacious to use vitamin D as an adjuvant therapy in the treatment of a number of infections.^[20,27,28]^ For instance, the trial by Cannell et al^[29]^ reported no common cold or influenza in participants after vitamin D supplementation. However, most studies suggested that vitamin D as an adjuvant therapy still needs to be explored and investigated.^[30,31]^ In our study, it was difficult to determine an accurate vitamin D dose in the treatment of OM. Most experts recommended a daily intake of 600 IU for all children.^[2,32,33]^ However, 2 important studies pointed out that the dosage of vitamin D supplementation depended on different serum 25(OH)D levels.^[2,34]^

This meta-analysis has several advantages. The first is that the likelihood of including all relevant published articles was improved via the comprehensive literature search. Secondly, the large sample size allowed us to quantitatively evaluate the association between serum vitamin D levels and both occurrence and development of OM. Therefore, our study provided a more robust assessment than those of any individual study. Next, the meta-analysis and systemic review included participants with a wide range of ages and all existing observational studies related to different types of OM, which allowed us to gain a comprehensive assessment of the association between vitamin D and incidence of different types of OM.

There are several limitations in current study: publication bias was not evaluated due to few studies investigating the relationship between serum vitamin D level and the risk of OM; the study used pooled data, which restricted a more detailed and comprehensive analysis; and several important confounders could not be adjusted, because few studies evaluated the association between serum vitamin D level and the risk of OM.

To sum up, the results of this study suggested that the lower serum 25(OH)D levels or vitamin D deficiency might play an important role in increasing the occurrence of AOM.
